# Research on the influence mechanism of exploitative leadership on employees' job insecurity from an observer's perspective

**DOI:** 10.3389/fpsyg.2026.1715349

**Published:** 2026-03-23

**Authors:** Can Li, Aoqi Li, Jinyu Liu, Chunyong Tang

**Affiliations:** 1 Party School of the CPC Fuxin Municipal Committee, Fuxin, China; 2 Marxist Studies Institute, LiaoNing Finance and Trade College, Huludao, China; 3 School of Economics and Management, Southwest Jiaotong University, Chengdu, China

**Keywords:** bystander effect, exploitative leadership, job insecurity, leader-member exchange, psychological contract breach

## Abstract

Exploitative leadership, as a negative leadership style, directly harms targeted subordinates while indirectly affecting bystanders. Moreover, unlike prior literature that has focused more on employees' moral outrage and schadenfreude toward such incidents. This study, grounded in Social Information Processing Theory (SIPT), shifts the focus to the bystander perspective, examining how witnessing such behavior leads to distinct perceptual changes among employees–specifically, job insecurity. A dual research design ensures external and internal validity: Study 1 employs a two-stage questionnaire survey with 230 Chinese employees, while Study 2 conducts a situational experiment involving 162 participants with work experience. Research results show that exploitative leadership significantly aggravates the work insecurity of bystanders, and the psychological contracts breach plays an intermediary role. The leader-member exchange (LMX) relationship regulates this impact: a high-quality LMX relationship can reduce individuals' susceptibility to exploitative leaders. This study introduces the perspective of bystanders and constructs a theoretical framework and clarifies how exploitative leaders can cause the occupational insecurity of bystanders' employees in specific situations. This broadens the research perspective on exploitative leadership and highlights its spillover effect on the organization. This helps enterprises recognize the profound impact of exploitative leadership, thus promoting the long-term development of enterprises.

## 1 Introduction

Exploitative leadership refers to a leadership style mainly for the purpose of promoting the leader's own interests, which is usually achieved by encroaching on the labor results of followers, putting pressure on and manipulating followers, overloading followers and hindering their development (Schmid et al., 2019). Although past research has widely documented the harmful effects of this behavior on the targeted subordinates, it not only affects the emotions and mental health of employees but also prompts them to adopt negative behaviors that are detrimental to the organization (Schmid et al., 2019; Guo et al., 2021, 2024a). However, the current understanding of exploitative leadership is mainly limited to the dual relationship of “leader-victim,” and there is a relative lack of research on the experience of bystanders.

Exploitative leadership can cause a series of negative effects on employees, such as counterproductive behavior and knowledge concealment (Guo et al., 2024b; Feng et al., 2022; Syed et al., 2021). However, this impact extends beyond targeted subordinates. When individuals witness such behavior as bystanders, they may be affected as well. For instance, abusive management and workplace bullying exert negative effects on observers (Dhanani and LaPalme, 2019; Priesemuth and Schminke, 2019; Kay et al., 2023; David et al., 2023). Yet exploitative leadership differs from these highly hostile leadership styles. Exploitative leadership typically exerts influence through subtle means, achieving objectives by deftly manipulating social relationships (Schmid et al., 2019). It is comparatively covert and less overtly hostile (Pircher Verdorfer et al., 2024). Yet precisely because of this subtlety, its cumulative impact on employees may become more profound. Therefore, exploring the bystander effects of exploitative leadership in the current context is essential.

The existing literature on the bystander effect in destructive leadership reveals a thought-provoking paradox. On one hand, research on abusive supervision indicates that witnessing others being mistreated triggers moral outrage and altruistic behavior in observers, who attempt to punish perpetrators and protect targeted subordinates (Priesemuth and Schminke, 2019; Kay et al., 2023; Chen and Liu, 2019). On the other hand, bystanders may feel satisfied or even experience schadenfreude (Mitchell et al., 2015; Li et al., 2019). However, in such an environment, employees tend to feel more helpless. This leads them to perceive potential threats. While David et al. (2023) identified that vicarious abuse can cause employees to perceive identity threats, their research lacks exploration of the deeper perceptions that emerge after such threats are perceived. Given that the perpetrator is a leader holding a position of power, this “power asymmetry” may prevent employees from responding effectively. In this context, employees acting as bystanders lack effective coping mechanisms. This fosters profound anxiety about workplace uncertainty, thereby intensifying job insecurity (Debus et al., 2014). Therefore, to address the deeper perceptual impacts of destructive leadership on bystanders, this study focuses on job insecurity. It explores bystanders' reactions after perceiving the threat of destructive leadership.

This study constructs a model based on Social Information Processing Theory (SIPT). According to SIPT, employees construct their perceptions of the organizational environment by interpreting key social cues within it, using these cues to predict potential future career trajectories (Salancik and Pfeffer, 1978). Witnessing unfair behavior as a bystander may trigger subsequent psychological and physiological stress responses (Dhanani and LaPalme, 2019). Such incidents directly induce psychological states in third parties, including anger (Mitchell et al., 2015) and work frustration (Harris et al., 2013). The persistence of these negative psychological states may damage employees' trust in the organization and eventually lead to the breakdown of the psychological contract breach. Therefore, this study proposes that witnessing a third party's exploitative leadership behavior will trigger the breakdown of the individual psychological contract breach, which will lead to work insecurity.

In addition, in addition to obtaining organizational information through observation, individuals can also establish communication relationships with leaders to gain more in-depth insights. As a common hierarchical dynamic within such relationships, the Leader-Member Exchange (LMX) represents the quality of the exchange established through interactions with leaders (Khorakian and Sharifirad, 2019). A high LMX elevates employees to the leader's “inner circle,” granting them preferential access to resources (Gottfredson et al., 2020). Consequently, these employees are more inclined to align with the leader, internalizing observed exploitative behaviors as strategic arrangements made for the sake of work. Employees in this state do not experience psychological contract breach when observing third parties subjected to exploitative leadership. Therefore, this study selects LMX as the moderating factor to investigate the boundary conditions of the observed exploitative leadership.

**Figure 1:**
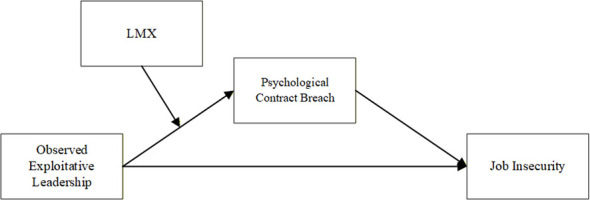
Research model.

In summary, this research aims to explore the impact of observing third-party exploitation by leaders on individual job insecurity, focusing on the mediating role of bystander psychological contract breach and the moderating role of LMX (see Figure 1). This study makes three key contributions. First, it researches the effects of exploitative leadership on witnesses from the bystander's perspective, breaking away from previous research that solely investigated the negative impacts of exploitative leadership from the victim's viewpoint (Schmid et al., 2019; Guo et al., 2021; Feng et al., 2022; Syed et al., 2021). These complements existing exploitative leadership literature, deepening our understanding of its consequences. It also responds to Schmid et al. (2019) call to examine how exploitative leadership, distinct from abusive management, produces similar yet nuanced effects. Second, grounded in SIPT, we reveal that negative leadership behaviors undermine bystanders' psychological contract breach, offering fresh insights for third-party perspective research. Finally, by introducing LMX as a moderator, this study further highlights the critical role of LMX in shaping bystanders' processing of negative leadership information. It also expands the boundaries of research on how third parties experiencing exploitative leadership impact employees from a bystander perspective.

## 2 Theoretical and hypothesis

### 2.1 Exploitative leadership

Exploitative leadership was formally defined and measured by Schmid et al. (2018). They describe it as an act in which leaders deceive, encroach upon and pressure their subordinates for personal purposes. Similar variables include abusive regulation and the Dark Triad. But they are essentially completely different. Abusive supervision is mainly manifested as persistent verbal or non-verbal hostility, such as public mockery, humiliation or exclusion, and its purpose often has the color of emotional venting or punishment (Tepper, 2000). In contrast, exploitative leadership is relatively covert and less hostile (Pircher Verdorfer et al., 2024). The Dark triplet represents a stable, cross-contextual malicious personality trait (Machiavellianism, narcissism and psychopathy), with the core lying in the individual's deeply rooted coldness and arrogance (Paulhus and Williams, 2002). Relevant studies have demonstrated that Machiavellianism and narcissism in the Dark Triad are important predictor variables of exploitative leadership (Schmid et al., 2018; Huang et al., 2023).

### 2.2 The impact of observed exploitative leadership on job insecurity

Leaders' uncivil behaviors occur within social contexts, where bystanders can detect social information that helps them understand their position within the group (Duffy et al., 2006). According to the SIPT, employees shape subsequent attitudes and behaviors by processing and interpreting information within the organization (Salancik and Pfeffer, 1978). When employees witness exploitative leadership as third-party observers, this observed uncivil behavior may be perceived more intensely than when experienced directly (Dhanani et al., 2018; David et al., 2023). This occurs because observers often assess potential threats within the environment from a detached perspective. Consequently, employees experiencing exploitative leadership as observers are also affected by its spillover effects.

First, exploitative leadership emphasizes leaders seizing personal gains at subordinates' expense, characterized by systematic appropriation of subordinates' time, energy, and outcomes (Schmid et al., 2019). Witnessing such behavior signals not merely an unfair incident but a fundamental rule governing organizational dynamics. This means that in this environment, it may be difficult for an individual's efforts to get the due reward, and it may even be stolen by others at any time. Such signs often undermine employees' awareness of organizational fairness and weaken their sense of control of the environment (Aquino et al., 2004). Therefore, employees are more likely to worry about their future situation, thus worrying about career development.

Secondly, when employees witness exploitative leadership behavior, their handling and interpretation of this information will enhance their sensitivity to negative signals in the organizational environment (Baumeister et al., 2001). When employees see that their colleagues are being treated unfairly, they tend to interpret it as a situational threat and assess their own vulnerability and potential risks accordingly (Reich and Hershcovis, 2015). This aggravates the employees' sense of uncertainty about the organizational environment. When employees learn that their colleagues have encountered exploitative leadership, they may worry that they will face similar treatment and regard this as a threat to their job security (Glambek et al., 2018). Work insecurity is the core cognitive manifestation of this perceived threat (Greenhalgh and Rosenblatt, 1984; De Witte, 2005). In addition, uncertainty usually triggers negative emotions such as anxiety and tension, which have been proven to be important psychological factors leading to job insecurity (Debus et al., 2014). Based on this logic, when employees observe that their colleagues are subjected to exploitative leadership, they are more likely to interpret it as social information that foreshadows their own risks, thus increasing job insecurity. Therefore, we hypothesize:

H1: Observed exploitative leadership positively correlates with job insecurity.

### 2.3 The mediating role of psychological contract breach

Psychological contract breach refers to the individual's belief that the organization has failed to fulfill certain obligations corresponding to its contribution in the psychological contract (Robinson and Rousseau, 1994). Psychological breach of contract has cognitive attributes, reflecting the psychological measurement of the individual's acquired obligations and committed obligations. Existing research mainly focuses on how psychological contracts are triggered and violated when employees feel unfair or lack of resources, and how this further affects their attitudes and behaviors (Tekleab et al., 2005). However, this view assumes that the psychological contract mainly comes from direct experience but ignores how employees' understanding of organizational exchange relationships is constantly updated and reconstructed in social situations. Therefore, this study proposes that from the perspective of bystanders, individuals will also feel insecure at work through the psychological contract breach.

According to SIPT, employees regard the experiences of other people in the organization as an important clue to judge “how the organization treats employees” (Salancik and Pfeffer, 1978). Witnessing uncivilized leadership behavior will significantly affect bystanders' perception of organizational fairness and values, which are the basis for their relationship with the organization (Dunford et al., 2015). When employees witness exploitative leadership behavior, such as encroaching on colleagues' work results or increasing workload, subordinates will think that they have been treated unfairly (Schmid et al., 2019). Therefore, the destruction of their expectations of the organization or superiors may lead to a feeling of betrayal and anger (Priesemuth and Schminke, 2019). If the organization fails to provide corresponding returns, employees may think that the organization or superiors have violated the expected exchange relationship (i.e., psychological contract), resulting in their unwillingness to fulfill their obligations to the organization (Tekleab and Taylor, 2003). Therefore, negative social information from the external environment will reshape employees' cognitive structure of organizational relationships, thus causing the psychological contracts breach.

When employees' perceived commitments or reciprocal rewards are broken (i.e., the psychological contract is violated), they will experience a decline in job satisfaction (Raja et al., 2004) and a decrease in trust in the organization, an increase in uncertainty and an increase in a sense of relative deprivation (Miner-Rubino and Reed, 2010; Lin et al., 2022). These cognitive and emotional reactions will further weaken employees' sense of control over their own work situation (Coyle-Shapiro et al., 2019), intensify their assessment of future environmental uncertainty, and ultimately show an increase in job insecurity. Therefore, this study posits that psychological contract breach serves as the key pathway through which observed exploitative leadership influences employee job insecurity. Therefore, we hypothesize:

H2: Psychological contract breach mediates the relationship between observed exploitative leadership and job insecurity.

### 2.4 The moderating role of leader-member exchange

Leader-Member Exchange (LMX) characterizes employees' interactions with their direct supervisors within an organization and the varying quality of social relationships formed through repeated interactions (Khorakian and Sharifirad, 2019). High-LMX “insiders” enjoy greater access to resources, receive more attention from leadership, and have increased promotion opportunities, forming social exchange relationships with leaders based on loyalty, mutual trust, and respect. Conversely, low-LMX “outsiders” possess only rights and obligations derived from employment contracts, maintaining economic exchange relationships with leaders characterized by lower levels of mutual trust (Gottfredson et al., 2020). Thus, LMX determines individuals' perceptions of the subordinate-leader relationship.

Based on Social Information Processing Theory (SIPT), individuals selectively process and interpret environmental cues by integrating their relational experiences with significant others in the context (Salancik and Pfeffer, 1978). For employees with high LMX, the strong trust foundation and emotional bonds with their leaders lead them to interpret leadership misconduct through situational or external attributions. For instance, they may attribute negative leadership behaviors to situational stress, task overload, or individual employee issues rather than inherent leadership flaws (Martinko et al., 2013). In other words, high LMX prompts observers to perceive exploitative leadership as a legitimate demand, thereby sustaining their belief in the organization's fulfillment capacity and reciprocity norms. In contrast, employees with low LMX, lacking a foundation of trust and emotional support with their leader, are more likely to interpret exploitative behavior as opportunistic leadership or even as a significant signal of organizational tacit approval of injustice (Dulebohn et al., 2012). Under this cognitive framework, bystanders are more likely to elevate such events into broader dissatisfaction with organizational exchanges. This strengthens their perception of the organization's failure to fulfill implicit commitments, thus triggering the psychological contract breach. It is evident that LMX mediates the process by which observed exploitative leadership influences psychological contract breach. Therefore, we hypothesize:

H3: LMX moderates the relationship between observed colleagues encountering exploitative leadership and psychological contract breach, weakening the positive correlation for individuals with high-quality LMX.

According to the SIPT, individuals' cognition and attitude toward the organizational environment are not formed in isolation, but are constructed through the continuous processing and interpretation of key social clues in the work environment (Salancik and Pfeffer, 1978). Based on the above reasoning, we can infer that when employees observe exploitative leadership behavior at work, this negative social message will be regarded as a signal about employee value and reciprocity norms transmitted by the organization. This will trigger a reassessment of the organization's commitment performance and may lead to the psychological contract breach. In turn, this will further reduce employees' expectations of future career stability and exacerbate their job insecurity. However, this path from “observed exploitative leadership” to “psychological contract breach” to “job insecurity” does not have the same impact on all employees. As a reflection of the quality of the exchange relationship between individuals and direct leaders, the exchange of leader members (LMX) relationship affects how employees screen and interpret social information. Employees with high LMX, respect and reciprocity are more likely to interpret observed exploitative behavior as situational pressure or expedient rather than a stable leadership trait or organizational position. This mitigates the impact of negative signals on the psychological contract. Conversely, employees with low LMX, lacking emotional support and a foundation of trust, are more likely to interpret such behaviors as signals of organizational-sanctioned injustice and relational imbalance. This intensifies perceptions of psychological contract breach and further amplifies pessimistic expectations regarding job stability and feelings of job insecurity. Accordingly, this study proposes that psychological contract breach mediates the relationship between perceived exploitative leadership and job insecurity (H2), and that LMX moderates the effect of perceived exploitative leadership on psychological contract breach (H3), thereby constituting a moderated mediation model: leadership-member exchange moderates the indirect effect of perceived exploitative leadership on employee job insecurity through the key mechanism of psychological contract breach.

H4: LMX moderates the indirect effect of observed exploitative leadership through psychological contract breach on job insecurity. Specifically, when LMX levels are higher, the positive indirect effect is weaker.

## 3 Overview of studies

We adopted complementary research methods to test the theoretical model through two studies. In Study 1, we collected questionnaire data from two-time points and tested all the hypotheses. Although this study allows us to test the model in the organizational environment and provide ideal external validity, it is essentially observational and may not lead to strong causal inferences. Therefore, we carried out follow-up experimental micro-story research and conducted a control test on key propositions. Specifically, in Study 2, we experimentally tested Hypothesis 1–4 by manipulating the observed exploitative leadership behavior and the LMX.

## 4 Study 1: method

This research employed an online questionnaire collection method, utilizing the Credamo platform for data gathering. To avoid homogenous bias, the research strictly adopted a two-stage lagged survey design. In the first stage (T1), employees collected 300 valid questionnaires, surveying third-party observations of exploitative leadership, leadership-member exchange (LMX), and relevant demographic information. One month later (T2), the same 300 employees who responded in Phase 1 were invited to assess their psychological contract breach and job insecurity. This phase yielded 230 valid responses, representing a 62.6% recovery rate.

### 4.1 Data collection and variable measurement

Since all scales used in this study are in English, they require translation into Chinese prior to use. To ensure rigor, we employed two approaches: First, for scales previously used in Chinese contexts, we referenced existing translations from those studies and adjusted accordingly. Second, after translation, we invited scholars in the field to review the materials, ensuring accuracy.

*Observed exploitative leadership*. This study adapted the 15-item exploitative leadership scale developed by Schmid et al. (2019) following the methodology used by David et al. (2023) to modify the alternative abuse scale. The scale uses a seven-point Likert-type scale. This scale consists of five dimensions: authentic selfish behavior, exerting pressure, stifling development, taking credit, and manipulative behavior. Representative items include statements such as “My supervisor views my colleagues as means to achieve personal goals” (Cronbach's α = 0.93).

*LMX*. We measured LMX using the seven-item scale developed by Graen and Uhl-Bien (1995). The scale uses a seven-point Likert-type scale. Representative items include statements such as “How well does your leader recognize your potential?” (Cronbach's α = 0.94).

*Psychological Contract Breach*. We measured psychological contract breach using the four-item scale developed by Robinson and Wolfe Morrison (2000). The scale uses a seven-point Likert-type scale. Representative items include statements such as “I feel a great deal of anger toward my organization” (Cronbach's α = 0.91).

*Job Insecurity*. We measured job insecurity using the four-item scale developed by Mauno et al. (2001). The scale uses a seven-point Likert-type scale. Representative items include statements such as “I'm worried I might get fired” (Cronbach's α = 0.90).

*Control*. In line with previous studies (Jin et al., 2016; Guo et al., 2024a), we selected supervisors' demographic characteristics, including gender, age, education, and organizational tenure, as control variables. Given that social ties between observers and targeted subordinates may interfere with their attribution of leadership exploitation and threat assessment (Duffy et al., 2006), this study follows prior research (Huang and Tang, 2024) by including collegial relationships as a control variable. This approach aims to eliminate interpersonal interference and more accurately identify the impact of leadership behaviors on observers' psychological contract breach. For measuring co-worker relationship, we selected the seven-item scale adapted by Menguc et al. (2016). Representative items include statements such as “I know how to support my colleague” (Cronbach's α = 0.91).

### 4.2 Analytical strategy

To test our hypotheses, we followed these steps: First, we conducted descriptive statistics using SPSS and calculated Pearson correlations to examine the initial bivariate relationships between variables. Second, we performed a series of confirmatory factor analyses in Mplus to evaluate the goodness of fit of the measurement model and assess the discriminant validity of the constructs. Third, we tested our hypotheses using hierarchical regression analysis in SPSS and conducted simple slope tests to illustrate the moderating effects. Finally, we used the PROCESS plugin in SPSS to compute confidence intervals and test the significance of the moderated mediation effects.

### 4.3 Confirmatory factor analyses

This study employed Harman's one-factor test to examine homogeneity of variance. Results indicate that the first principal component obtained without rotation accounted for 32.39% (cumulative 73.46%), which did not exceed 40%. It is proved that the data of this study do not have CMB. Prior to the hypotheses testing, we conducted a series of confirmatory factor analyses (CFA). The analysis results are shown in Table 1. We compared a single-factor model, a two-factor model, a three-factor model, as well as a four-factor model with all the five distinct constructs. Only the fit indexes of the four-factor model are acceptable (χ^2^/*df* = 2.55, CFI = 0.92, TLI = 0.91, RMSEA = 0.08, SRMR = 0.04), supporting discriminability of this data structure. To address potential common method bias (CMB) concerns, we implemented a latent method factor approach (Podsakoff et al., 2003). The fit indicators (χ^2^/*df* = 2.33, CFI = 0.94, TLI = 0.93, RMSEA = 0.07, SRMR = 0.03) demonstrated enhanced fit statistics. We compared the standardized factor loadings from the measurement model estimated with and without the inclusion of a common latent factor (CLF). The results showed that the differences in standardized loadings were uniformly small, with none exceeding the recommended threshold of 0.20. Given that multiple techniques were used to avoid CMB during data collection (e.g., using mixed questionnaire items, and distributing questionnaires at two time points) and that the baseline model met the recommended fit standards, we concluded that CMB was not an issue in this study (Podsakoff et al., 2003). Multicollinearity diagnostics indicated all variance inflation factors (VIFs) <5, thereby confirming the lack of collinearity issues.

**Table 1:** Measurement model comparison (*N* = 230).

**Model**	**χ^2^**	**df**	**χ^2^** **/*df***	**CFI**	**TLI**	**RMSEA**	**SRMR**
Four-factor Model (O-EL, LMX, PCB, JI)	987.64	388	2.55	0.92	0.91	0.08	0.04
Three-factor Model (O-EL+LMX, PCB, JI)	1,377.79	402	3.43	0.87	0.86	0.11	0.05
Two-factor Model (O-EL+LMX+PCB, JI)	1,444.10	404	3.57	0.86	0.85	0.11	0.05
One-factor Model (O-EL+LMX+PCB+JI)	1,692.97	405	4.18	0.83	0.82	0.12	0.05

O-EL, Observed Exploitation Leadership; LMX, Leader-Member Exchange; PCB, Psychological Contract Breach; JI, Job Insecurity.

### 4.4 Descriptive statistics

Table 2 presents the descriptive statistics (Means and Standard Deviations), and correlations for the variables. Consistent with our arguments, observed exploitation leadership is significantly positively correlated with psychological contract breach (*r* = 0.75, *p* < 0.01) and job insecurity (*r* = 0.83, *p* < 0.01). Psychological contract breach is significantly positively correlated with job insecurity (*r* = 0.74, *p* < 0.01). Additionally, the moderating variable, LMX, is significantly positively correlated with psychological contract breach (*r* = 0.77, *p* < 0.01). These correlations provide preliminary support for the hypotheses of this study.

**Table 2:** Descriptive statistics.

**Variable**	**M**	**SD**	**1**	**2**	**3**	**4**	**5**	**6**	**7**	**8**	**9**
1.Sex	0.43	0.50	1								
2. Age	1.76	0.85	−0.06	1							
3. Organizational tenure	2.80	0.87	−0.03	0.86^**^	1						
4. Education	3.20	0.57	−0.07	−0.04	−0.05	1					
5. Co-worker relationship	5.60	0.93	−0.00	0.08	0.1	0.07	1				
6. Observed exploitation leadership	2.81	1.34	0.08	−0.05	−0.12	−0.15^*^	−0.72^**^	1			
7. LMX	5.40	1.16	−0.09	0.12	0.15^*^	0.10	0.81^**^	−0.87^**^	1		
8. Psychological contract breach	2.13	1.17	0.14^*^	−0.15^*^	−0.15^*^	−0.12	−0.55^**^	0.75^**^	−0.77^**^	1	
9. Job insecurity	2.55	1.28	0.14^*^	−0.18^**^	−0.26^**^	–.10	−0.71^**^	0.83^**^	−0.84^**^	0.74^**^	1

^*^p < 0.05, ^**^p < 0.01.

### 4.5 Hypotheses testing

We used SPSS to conduct hierarchical regression analyses to test our hypotheses, with the results summarized in Table 3. Model 6 shows that observed exploitation leadership has a significant positive effect on job insecurity (β = 0.62, *p* < 0.001), supporting Hypothesis 1.

**Table 3:** Results of hypothesis testing.

**Variable**	**Psychological contract breach**				**Job insecurity**		
	**M1**	**M2**	**M3**	**M4**	**M5**	**M6**	**M7**
Sex	0.12^*^	0.07	0.04	0.04	0.13^**^	0.08^*^	0.07
Age	−0.09	−0.22^*^	−0.17^*^	−0.20^**^	0.13	0.02	0.08
Organizational tenure	−0.02	0.14	0.12	0.20^**^	−0.30^***^	−0.17^*^	−0.21^**^
Education	−0.08	−0.01	−0.02	−0.01	−0.06	0.01	0.01
Co-worker relationship	−0.53^***^	−0.02	0.21^**^	0.09	−0.68^***^	−0.25^***^	−0.24^***^
Observed exploitation leadership		0.74^***^	0.36^***^	0.29^***^		0.62^***^	0.44^***^
Psychological contract breach							0.26^***^
LMX			−0.62^***^	−0.13			
Exploitation leadership ^*^ LMX				−0.55^***^			
F-value	21.12	49.18	54.64	68.71	54.22	100.28	99.20
R^2^	0.33	0.58	0.64	0.72	0.56	0.74	0.77
△*R*^2^	0.32	0.57	0.63	0.71	0.55	0.73	0.76

^***^p < 0.001, ^**^p < 0.01, ^*^p < 0.05.

Models 2 reveal that observed exploitation leadership significantly increases psychological contract breach (β = 0.74, *p* < 0.001). Models 7 demonstrate that when adding psychological contract breach to Models 6, the impact of observed exploitation leadership on job insecurity remains significant (β = 0.44, *p* < 0.001). Psychological contract breach effect on job insecurity significant (β = 0.26, *p* < 0.001), indicating that psychological contract breach partly mediates the relationship between observed exploitation leadership and job insecurity. This supports Hypotheses 2.

To enhance the statistical power of the mediation analysis, we further applied the Bootstrap method. The results indicate that the indirect effect of observed exploitation leadership on job insecurity through psychological contract breach is 0.128, 95% CI = [0.070, 0.202], which does not include zero. Further confirming Hypotheses 2.

**Figure 2:**
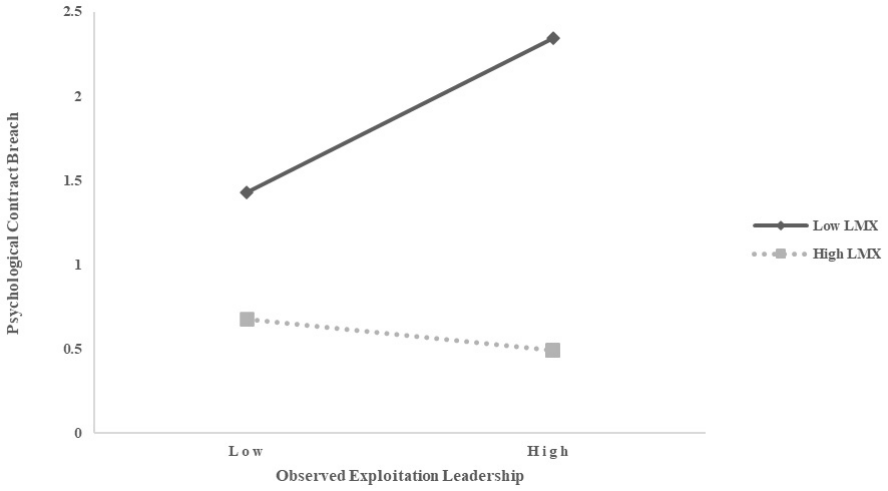
Interaction of exploitation leadership and LMX.

To mitigate the adverse effects of multicollinearity, this study centered on the variables of observed exploitation leadership and LMX and created interaction terms. As shown in Models 3 and 4 in Table 3, the interaction between observed exploitative leadership and LMX was negatively and significantly associated with psychological contract breach (β = −0.55, *p* < 0.001). These findings support Hypotheses 3. To better understand the mechanisms underlying these moderation effects, we conducted simple slope analyses and plotted the moderation effects, as illustrated in Figure 2.

We used the PROCESS macro in SPSS to examine the moderated mediation effects. In Table 4, under the high LMX condition, the indirect effect was not significant (Indirect Effect = 0.006, SE = 0.044, 95% CI [−0.079, 0.101]). Under the condition of low LMX, the indirect effect was significant (Indirect Effect = 0.156, SE = 0.052, 95% CI [0.048, 0.255]). The difference between the two conditions was also significant (Indirect Effect Difference = −0.075, SE = 0.026, 95% CI [−0.124, −0.022]), supporting Hypothesis 4.

**Table 4:** Analysis of moderated mediation effect.

**Variable path**	**Moderator**	**Estimate**	**SE**	**95%CI**	
O-EL → PCB → JI	High	0.006	0.044	−0.079	0.101
	Low	0.156	0.052	0.048	0.255
	Diff	−0.075	0.026	−0.124	−0.022

Bootstrapping = 5,000.

## 5 Study 2: method

The purpose of this research is to use a scenario experiment to examine the interaction between observed exploitative leadership and LMX on the impact of psychological contract breach.

### 5.1 Sample and procedure

Participants were recruited via the Credamo platform, a professional data integrated service provider widely utilized in organizational behavior research. Before the scenario experiment, participants were informed that the study aimed to understand workplace interpersonal dynamics and that their participation was entirely voluntary. They were granted the right to withdraw from the study at any point without any negative consequences. Ultimately, a total of 162 subjects were recruited for this experiment. The average age of participants was 31.59, with an average work experience of 8.13 years. Males accounted for 50% of participants, and the vast majority (67.9%) were employees of private enterprises. 81.5% of participants held a bachelor's degree.

The research design followed the ethical standards of the Institutional Review Board (IRB) and the Declaration of Helsinki. All data collection was conducted anonymously. No personally identifiable information (e.g., names, ID numbers, or specific company names) was collected. All raw data were stored in encrypted files accessible only to the research team and used solely for academic purposes. As a token of appreciation for their time and contribution, participants who completed the experiment received 5 RMB incentive through the platform's payment system. This compensation was predetermined and was not contingent on the nature of their responses to ensure data objectivity.

A 2 (observed third-party exploitation-type leadership: high vs. low) × 2 (LMX: high vs. low) between-subjects experimental design was employed, randomly assigning participants to four groups. Participants were instructed to imagine themselves as Zhang San, the protagonist in the material.

Your name is Liu Bo, a frontline employee at Company A where you have worked for some time. Working alongside you in the same team are your colleagues Xiao Li and Xiao Wang, while Zhang Qiang serves as your team leader. Zhang Qiang has been your supervisor since you joined the company, and you have known him for a long time.

*Observed Exploitative Leadership*. Based on experimental materials adapted from Huang and Tang (2024), this research further adapts the Exploitative Leadership Scale developed by Schmid et al. (2019). The Strong Relationship Group material states: “However, your supervisor frequently treats your colleague Xiao Li as a means to achieve his personal goals, often derives personal benefits from Xiao Li's work outcomes, and frequently increases Xiao Li's workload for his own advantage. On one occasion, you even overheard him attempting to drive a wedge between you and Xiaoli. ‘The weak relationship group received': However, your supervisor never treats employees as means to achieve personal goals, never derives personal benefit from employees' work outcomes, and frequently considers everyone's workload, avoiding excessive demands while actively fostering harmonious relationships within the team.” Using the same 15-item scale adapted from Schmid et al. (2019) as in Study 1, we tested manipulation validity (Cronbach's α = 0.919). Results confirmed the validity of this manipulation [M_High_ = 5.89, SD_High_ = 0.41; M_Low_ = 1.88, SD_Low_ = 0.50; *t*_(160)_ = 56.29, *p* < 0.001].

*LMX*. Draws on Tröster and Van Quaquebeke (2021) scenario wording for material composition. The strong relationship group material reads: “When you encounter problems at work, Zhang Qiang always helps you resolve them. If you make a mistake, he will ‘bail you out' at the expense of others. Moreover, he deeply understands your work challenges and needs. Your relationship is strong, and if you had to describe it, you might say it's highly effective. “The weak relationship group received:“ Your relationship with Zhang Qiang is poor. When you encounter problems at work, he rarely helps resolve them; yet if you make a mistake, he doesn't ‘bail you out' at others' expense. Furthermore, he doesn't understand your work issues or needs very well. Your relationship is not good. If you had to describe your relationship with him, you might say it is not very effective.” The same seven-item scale developed by Graen and Uhl-Bien (1995) used in Study 1 (Cronbach's α = 0.908) was employed. Results indicate the manipulation was effective [M_High_ = 5.89, SD_High_ = 0.53; M_Low_ = 2.06, SD_Low_ = 0.68; *t*_(160)_ = 39.95, *p* < 0.001].

### 5.2 Result

To test our hypotheses, we employed analysis of variance (ANOVA) with observed exploitative leadership (O-EL) (high vs. low) and leadership-member exchange (LMX) (high vs. low) as two factors, and psychological contract breach and job insecurity as dependent variables. Our results revealed significant main effects of observed exploitative leadership on both psychological contract breach and job insecurity: Participants exposed to high O-EL (M = 6.06, SD = 0.61) reported significantly higher psychological contract breach tendencies than those exposed to low O-EL (M = 4.98, SD = 1.60), *F*_(1, 249)_ = 96.55, *p* < 0.001; and high O-EL participants (M = 3.26, SD = 1.67) reported significantly higher job insecurity than the low O-EL group (M = 2.86, SD = 1.28), *F*_(1, 249)_ = 11.32, *p* < 0.001, supporting Hypothesis H1 and consistent with research 1 findings.

**Figure 3:**
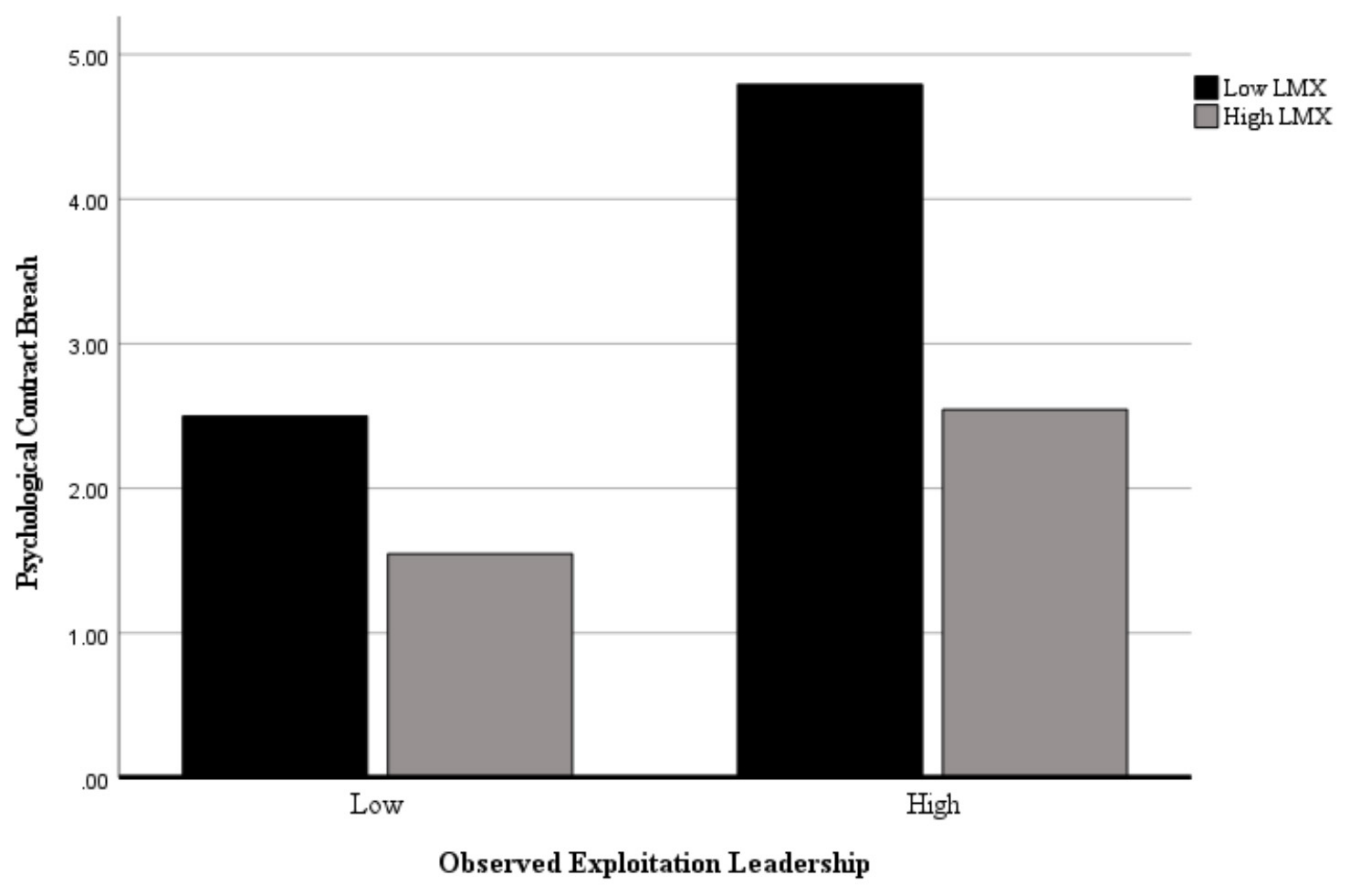
The moderation effect of LMX.

Furthermore, we observed a significant interaction between O-EL and LMX on psychological contract breach [*F*_(1, 158)_ = 14.03, *p* < 0.001]. Simple effect analysis (see Figure 3) revealed that under high LMX conditions, participants in the high O-EL group (M = 2.65, SD = 0.20) reported significantly higher psychological contract breach tendencies than those in the low O-EL group (M = 1.54, SD = 0.20), *F*_(1, 158)_ = 15.64, *p* < 0.001. Under low LMX conditions, participants in the high O-EL group (M = 4.98, SD = 0.19) reported significantly higher psychological contract breach tendencies than those in the low O-EL group (M = 2.41, SD = 0.20), with increased significance: *F*_(1, 158)_ = 88.60, *p* < 0.001. Consistent with Study 1, these findings again support H3.

## 6 Discussion

This study examines the mechanisms and boundary conditions through which observed exploitative leadership influences employees' job insecurity. Based on SIPT, we conducted two studies: scenario experiment and multi-wave questionnaire surveys. Study 1 provided high external validity through a follow-up questionnaire survey of 230 Chinese employees. Study 2, on the other hand, enhanced internal validity through situational simulation experiments and clarified the causal relationships among variables. The results of both studies show that there is a positive correlation between observed exploitative leadership and job insecurity, with psychological contract breach mediating this relationship. Furthermore, LMX not only mitigates the impact of observed exploitative leadership on psychological contract breach but also weakens the indirect effect of observed exploitative leadership on job insecurity via psychological contract breach. Theoretical and practical implications of these findings are discussed below.

### 6.1 Theoretical contributions

Our findings contribute to the existing literature in several keyways. First, the article reveals the spillover effects of exploitative leadership on bystanders, thereby broadening the research perspective on exploitative leadership. Past research on exploitative leadership has predominantly focused on the exploited individuals themselves (Schmid et al., 2019; Guo et al., 2021; Feng et al., 2022; Syed et al., 2021), while research on spillover effects remains relatively scarce. Consequently, whether and when bystanders recognize exploitative leadership remains under-explored. This perspective merits attention, as bystanders' attributions determine their perceived severity of events, their relationships with the focal parties, and group climate (Ashburn-Nardo et al., 2008; Jensen and Raver, 2021). Although existing research has confirmed the spillover effects of abusive leadership (David et al., 2023), exploitative leadership differs fundamentally from abusive interpersonal harm and verbal aggression in that it achieves private gain by appropriating employees' outcomes and controlling their actions (Schmid et al., 2019). Exploitative leadership is more subtle and covert (Pircher Verdorfer et al., 2024) yet precisely because of this, its spillover effects warrant greater attention. Our study fills a gap in research examining exploitative leadership from a bystander perspective, enriching the third-perspective literature on this phenomenon.

Second, we extend the spillover effects of exploitative leadership from a social information processing perspective. Witnessing exploitative leadership leads to individual psychological contract breach among bystanders, which further increases psychological uncertainty. The research findings contribute to expanding the theoretical perspective on exploitative leadership, deepen understanding of its spillover effects, and help unlock the internal “black box” mechanisms of its influence.

Finally, this study demonstrates the moderating role of LMX at the level of hierarchical relationships. Current research exploring the effects of the bystander effect has focused on boundary factors related to individual traits and environmental factors (David et al., 2023; Priesemuth and Schminke, 2019; Kay et al., 2023). This has largely overlooked the critical variable of interpersonal relationships, particularly those involving the central figure of the event—the leader. Therefore, from the perspective of LMX, this study further reveals the mechanism of how the observed exploitative leadership affects the third-party employees' job insecurity.

### 6.2 Management implications

The inspiration of this study on management practice is as follows: First, the research results show that exploitative leadership will cause bystanders to feel insecure at work. Therefore, managers should re-evaluate the impact of their negative behavior on employees. This impact affects not only exploited employees, but also bystanders. On the one hand, exploited employees may show negative behavior (Guo et al., 2021; Feng et al., 2022; Syed et al., 2021); on the other hand, bystanders will also have negative reactions. This hinders the formation of active mutual assistance between employees within the organization, which is not conducive to the long-term development of the organization.

Secondly, research shows that exploitative leadership behavior is a negative “social message,” which will trigger a psychological sense of contravention of onlookers, which will aggravate their insecurity at work. Therefore, enterprise managers should establish a transparent and fair communication mechanism, clarify the mutual commitment between the organization and employees, and ensure that employees' contributions can get reasonable institutional returns. At the same time, the human resources department should strengthen the ethical supervision of the behavior of middle managers. By establishing complaint channels or carrying out regular leadership style assessments, exploitative behavior can be detected and corrected in a timely manner. This method can prevent the spread of negative social information within the organization from the source, to maintain the psychological contract stability and organizational trust of bystanders.

Finally, this study reveals the regulatory role of LMX. The research results show that under high LMX conditions, the negative impact of leadership style on employees as bystanders will be reduced. Therefore, organizations should provide managers with training on how to build positive relationships with subordinates. This method can improve the quality of direct communication between employees and leaders, to cultivate a team with high-quality interactive relationships.

### 6.3 Research limitations and prospects

Although this research has contributed to the field of literature, there are also some limitations, which provide opportunities for future research. First, in terms of research methods, although this study adopts scenario experiments and measures conventional variables at two points in time to verify the causality and correlation between variables, all measurement data depends on the self-assessment of employees. Therefore, future research can consider using mutual evaluation between leaders and subordinates to collect data. Multi-source research design and longitudinal research design can provide more comprehensive data support. In addition, since the scenario experiment cannot guarantee the emotional stability of the participants during the experiment, it may affect the subsequent results. Future research can design laboratory experiments to maximize the emotional stability of participants.

Secondly, the samples of this study are taken from the background of Chinese culture, and the samples are relatively homogeneous. Future research can select employees from different cultural backgrounds as research subjects. This will help improve the repeatability of the bystander effect of exploitative leadership behavior in cross-cultural contexts. In addition, comparing the reactions of bystander employees from different cultural backgrounds to exploitative leadership behaviors can further reveal the unique impact of these behaviors, thus enriching the research on the bystander effect of exploitative leadership behavior.

Finally, in the study of the bystander effect, our research framework and many existing studies keep variables at the same level—that is, cognition, behavior, etc. at the individual level (David et al., 2023; Priesemuth and Schminke, 2019; Kay et al., 2023). This method lacks a deeper understanding of the bystander effect. Future research may consider constructing multi-level models to explore how variables at the leadership or team level are influenced, thereby broadening the scope of the bystander effect's impact.

## Data Availability

The raw data supporting the conclusions of this article will be made available by the authors, without undue reservation.

## Appendix 1. Scale items

(1) **Observed Exploitation Leadership**

My supervisor assumes that my colleagues' work achievements can be used for his or her personal gain.

My supervisor views my colleagues as means to achieve personal goals.

My supervisor prioritizes achieving his or her own objectives over my colleagues' needs.

My supervisor pressures my colleagues to meet his or her targets.

My supervisor increases my colleagues' workload to meet his or her goals without considering their needs.

When assigning new tasks, my supervisor fails to consider my colleagues' existing workload.

My supervisor assigns tedious tasks to my colleagues whenever it benefits him or her.

My supervisor denies my colleagues opportunities for professional growth because his or her own goals take precedence.

My supervisor assigns mundane, routine tasks to my colleagues whenever it serves his or her interests.

My supervisor exploits my colleagues' work to gain recognition.

My supervisor claims team achievements as his or her own.

My supervisor exploits my colleagues' work for personal gain.

My supervisor sows discord between my colleagues and me to achieve his/her objectives.

My supervisor manipulates my colleagues to advance his/her own goals.

My supervisor will not hesitate to manipulate or deceive my colleagues to achieve his/her objectives.

(2) **LMX**

Do you know where you stand with your leader..do you usually know how satisfied your leader is with what you do?

How well does your leader understand your job problems and needs?

How well does your leader recognize your potential? Regardless of how much formal authority he/she has built into his/her position, what are the chances that your leader would use his/her power to help you solve problems in your work?

Again, regardless of the amount of formal authority your leader has, what are the chances that he/she would “bail you out,” at his/her expense?

I have enough confidence in my leader that I would defend and justify his/her decision if he/she were not present to do so?

How would you characterize your working relationship with your leader?

(3) **Psychological Contract Breach**

I feel a great deal of anger toward my organization.

I feel betrayed by my organization.

I feel that my organization has violated the contract between us.

I feel extremely frustrated by how I have been treated by my organization.

(4) **Job Insecurity**

I'm worried I might get fired.

The thought of being fired really scares me.

I'm not worried about the future of this job.

I'm confident this job will last a long time.

(5) **Co-worker Relationship**

I know how to support my colleagues.

My colleagues understand my work challenges and needs.

My colleagues recognize my potential at work.

My colleagues help me overcome work difficulties.

My colleagues will sacrifice their own interests to help me out of work predicaments.

Whether my colleagues are present or not, I will uphold and defend their decisions.

I have a good working relationship with my colleagues.
